# Business model innovation: a review of the process-based literature

**DOI:** 10.1007/s10997-021-09590-w

**Published:** 2021-08-14

**Authors:** Daniela Andreini, Cristina Bettinelli, Nicolai J. Foss, Marco Mismetti

**Affiliations:** 1 University of Bergamo, Bergamo, Italy; 2 Department of Strategy and Innovation, Copenhagen Business School, Frederiksberg, Denmark

**Keywords:** Business model innovation process, Systematic literature review, Processes, Process-based business model innovation, BMI process interconnection

## Abstract

Research on business model innovation (BMI) processes is blossoming and expanding in many directions. Hence, the time is ripe to summarize and systematize this body of knowledge for the benefit of current and future BMI scholars. In this article, we take stock of the current literature to clarify the concept of a BMI process, develop a categorization scheme (a “BMI process framework”), and discuss future research possibilities. Building on a systematic literature review of 114 papers, our categorization delineates different types of BMI processes and corresponding sub-processes. Moreover, we develop a framework that illustrates how BMI processes are interrelated and interconnected. Finally, we identify the main process-related research gaps in BMI research and provide directions for future research that emerge from our categorization and discussion.

## Introduction

Scholars are increasingly making use of the notion of business model innovation (BMI) to frame and analyze complex firm-level issues that have a strategic and systemic dimension (Foss & Saebi, 2018). BMI is viewed as playing a pivotal role in firm success and performance (e.g., Cucculelli & Bettinelli, 2015). Most contributions have focused on what a BMI is in terms of its content, which is typically conceptualized as a new configuration of a company’s value proposition, value-capture activities, and/or value chain organization (Teece, 2010). More recently, however, the idea has developed that BMIs can also be understood in *process* terms (Wirtz et al., 2016). While there has been an increase in interest in BMI processes, the meaning of a BMI process varies across studies, and the nature of the construct is still fragmented and ambiguous. Some initial attempts have been made (Wirtz & Daiser, 2018), but a broader and more systematic review is still needed to integrate and synthesize the rather diverse literature on BMI processes.

To fill this gap, we selected 114 papers published between 2001 and 2020 and carried out a systematic literature review to shed light on the different categories of BMI processes and delineate the mechanisms underpinning them. We start from an understanding of process theory as the rigorous and systematic description of the “generative mechanisms or set of mechanisms at work … and their resulting outcomes” (Cornelissen, 2017, p. 5). On that basis, we identify process theory in the management literature that sees BMI as, among other things, a multilevel phenomenon that is guided by an overarching logic (Amit & Zott, 2001; Chesbrough & Rosenbloom, 2002; Teece, 2010) and can “coordinate and manage interrelated sets of activities performed by different actors” (Leischnig et al., 2017, p. 2).

In so doing, we first develop a categorization of different types of BMI processes (i.e., generative cognition processes, knowledge-shaping processes, strategizing processes, value creation processes, and evolutionary learning processes) to define them and their related sub-processes. Second, we develop a framework showing how the various BMI processes are distinct from each other but also interrelated and interconnected via evolutionary learning processes. Third, we propose an agenda for future investigations within the main BMI processes we identified in our systematic literature review, along with methodological challenges that can represent interesting opportunities for future research endeavors.

In sum, our article identifies different streams in BMI process research and integrates the various conceptualizations of BMI processes to gain a better understanding of this multifaceted construct. Doing so not only helps future researchers position their work within the broader literature but also facilitates the identification of the major actors (e.g., top management team; external stakeholders) involved in the process being investigated, as well as the nature of this process. We elaborate on these ideas in a research agenda whose aim is to generate novel and interesting avenues that subsequent studies can use to further improve theory development on BMI processes.

## Methods

### Procedure

To ensure reliable, replicable, and synthetic results (Denyer & Tranfield, 2009; Tranfield et al., 2003), we undertook a systematic literature review that follows a more rigorous, clear, and transparent method for data collection and analysis than other review methods (e.g., narrative literature reviews). Appendices 1 and 2 provide a detailed description of the procedure (i.e., planning, execution, and reporting) we used, as suggested by Tranfield et al. (2003).

We looked for papers on *Ebsco*, which is among the most prominent bibliographical databases scholars use to conduct literature reviews (e.g., Abatecola et al., 2013; Franco‐Santos & Otley, 2018). The time frame was limited to articles published before December 2020, with no initial time boundaries. Following Skjølsvik et al. (2017) and Franco‐Santos and Otley (2018), we filtered the results for journals listed in the Academic Journal Guide (2018) by the Chartered Association of Business Schools (i.e., the ABS Academic Journal Guide). The concept of BMI has been developed and applied in many different management research fields, including marketing, entrepreneurship, strategy, technology, operations management, and organizational studies (Foss & Saebi, 2017). Different fields are likely to conceptualize and theorize the BMI phenomenon in various ways and, therefore, use varying terminology. For this reason, we adopted search strings (research keywords) according to the objectives of the systematic review, as shown in Appendix 1, while the results of the procedure are reported in Appendix 1, Table A1. Following Thorpe et al. (2005), we read the titles, abstracts, and introductions of the papers and classified them into three categories: “A” included studies that were definitely relevant, “B” included studies whose relevance was initially unclear, and “C” included studies that were not relevant. As shown in Appendix 1 (point D), we followed an interactive process for inclusion in and exclusion from categories A and B, which resulted in a final sample of 114 papers.

### Analysis

Our analysis involved conducting both descriptive and interpretative investigations; see Appendix 2 (Thorpe et al., 2005; Tranfield et al., 2003). In particular, we recorded the definitions and boundaries of the BMI process concept as expressed by the author(s) of each paper.

We performed an interpretive analysis of the selected papers by following the procedures that Braun and Clarke (2006) suggest for thematic analysis. Thematic analysis is a method for identifying, analyzing, and reporting patterns (themes) derived from data (Braun & Clarke, 2006). In a systematic literature review, the themes are the main concepts on which an article is built, and they are expressed in the research questions, definitions, measurements, and results (Jones et al., 2011; Thorpe et al., 2005). In contrast with content analysis, themes are not identified as “the most representative” or “the most frequently mentioned” concepts, but they do capture important concepts relating to the research objectives (Ryan & Bernard, 2000).

We conducted an inductive data-driven thematic analysis without any pre-existing theoretical or coding frame. This approach requires reading and re-reading data in iterative cycles to identify process-based themes. The themes identified from the first analysis were diverse; thus, following Jones et al. (2011), we applied an interactive process of theme accordance and categorization to ensure consistency within and across theme categories. In this way, we identified classifications containing process-based themes and checked for duplication and redundancy at each level (Jones et al., 2011; Noy & McGuinness, 2001). Appendix 2 shows the steps followed to validate the thematic and the ontological analysis.

## Insights from the review

The early process-based definitions of BMI dealt with deliberate managerial processes aimed at innovating the core activities of companies (e.g., Demil & Lecocq, 2010; Morris et al., 2005; Nenonen & Storbacka, 2010; Zott & Amit 2007). More recently, Wirtz et al. (2016, p. 4) defined BMI in explicit process terms as “the design process for giving birth to a fairly new business model on the market, which is accompanied by an adjustment of the value proposition and/or the value constellation and aims at generating or securing a sustainable competitive advantage.” Foss and Saebi (2017, p. 216) define BMI as “designed, novel, and non-trivial changes to the key elements of a firm’s business model and/or the architecture linking these elements.” In particular, Foss and Saebi (2017) identify four different types of BMIs, which relate to *modular*, *architectural, radical, and incremental* BM changes. The first (modular BMI) is innovation that is related to specific BM sources and components of value. It emphasizes changes in the single components of a BM, such as entering new industries, changing the revenue model and/or redefining organizational boundaries, and innovating technologies, value networks, and financial hurdle rates. The second type of BMI (architectural BMI) examines new ways of linking activities or governing activities, as well as novel links between BM components. The third and the fourth types of BMI relate to their degree of novelty for the company and the industry (Foss & Saebi, 2017).

Building on these definitions, we suggest that BMI is a set of deliberate acts that managers and entrepreneurs perform over time to change the BM components and architecture in a consistent and innovative way (Foss & Saebi, 2017). The ultimate aim is to gain a strategic advantage (Amit & Zott, 2015).

In this stance, these definitions are in line with process theory; for instance, Van de Ven (1992; p.170) defines a process as a “sequence of events or activities that describes how things change over time, or that represents an underlying pattern of cognitive transitions by an entity in dealing with an issue,” where entities can be individuals, a workgroup, or the overall organization. Thus, the process theory developed by Van de Ven (1992) and Van de Ven & Poole (1995), inspired our categorization and the construction of our framework in two ways. First, the BMI processes are human-driven and implemented across different levels by people, which implies we have to consider processes not only at the corporate level but also from other entities’ perspectives. Second, process theory considers processes as dynamic activities that are interconnected and interrelated between entities, activities, and other processes (Nailer & Buttriss, 2020; Rescher, 1996). For these reasons, to understand processes, it is important to consider and identify patterns of interrelations and connections.

### Main characteristics of the papers in the sample

**Fig. 1:**
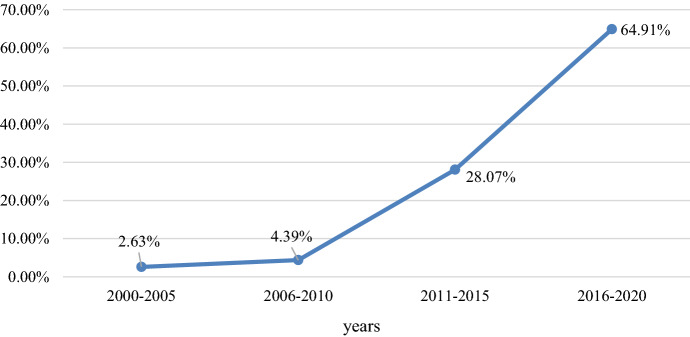
Overview of articles over time

The literature relating to BMI processes has developed rapidly in recent years. The first contributions emerged in the early 2000s (e.g., Chesbrough, 2010; Demil & Lecocq, 2010; Malhotra, 2002). As shown in Fig. 1, 28% of the articles we identified were published between 2011 and 2015. Since 2016, there has been a significant increase in the number of papers (65% of the papers in our sample) focusing on BMI processes, but they have mostly been neglected by many recent literature reviews (e.g., Foss & Saebi, 2017), which cover a different period. Thus, the review of articles shows that BMI processes are not only a recent area of focus in the BMI literature but also rapidly expanding (e.g., Laïfi & Josserand, 2016).

**Fig. 2:**
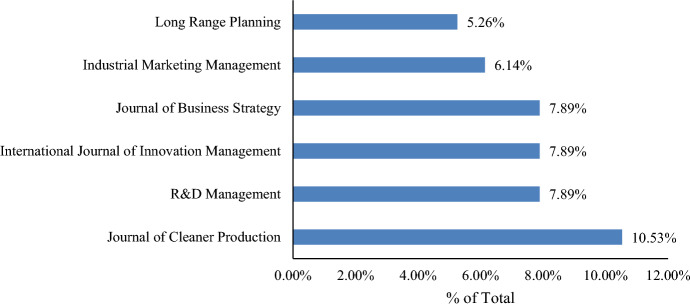
Most prolific journals

The six most prolific journals account for more than 45% of the 48 publications reviewed (Fig. 2). It is worth noting that the most prolific journals are not always the most established ones—that is, their impact factor is not necessarily the highest and they are relatively new. Thus, probably because it is an emerging theme, it seems that the BMI process, at least for now, has been better received in emerging publication outlets than in more established journals.

Our review highlights that 82 out of the 114 studies are based on qualitative methods, and eight are quantitative studies. Only two articles adopted both qualitative and quantitative methods: Cortimiglia et al. (2016) combined a quantitative survey with qualitative multiple case studies, while Eppler and Hoffmann (2012) mixed a survey with qualitative observations. The few remaining studies are conceptual articles (e.g., Andreassen et al., 2018; Remane et al., 2017) or offer a partial review of the literature where the focus is on the BM itself, and the processes of BMI are considered but treated as side aspects and not as the main focus of the review (e.g., Schneider & Spieth, 2013).

Moreover, 65% of the papers in our review consider two or more process types simultaneously (29% considered two processes, 29% three processes, and 7% four processes simultaneously); 35% focused only on one type of process. The type of approaches researchers can use to investigate BMI processes can be linear, recursive, parallel, or conjunctive (Van de Ven & Poole, 1995). Linear approaches represent a process as a “sequence of prescribed stages” (Van de Ven & Poole, 1995, p. 514), where each step is presented in a linear, unidirectional progression. By contrast, recursive approaches represent processes as cycles that continuously adapt via feedback loops (Cloutier & Langley, 2020). Parallel approaches have at least two linear interconnected process paths (Cloutier & Langley, 2020), while conjunctive approaches seek “to make connections between diverse elements of human experience through making those distinctions that will enable the joining up of concepts normally used in a compartmentalized manner” (Tsoukas, 2017, p. 148). In our review, we found that most of the papers did not have an explicit preference for one type of approach over another. However, 30 of the 107 studies present the BMI processes with a linear process style (e.g., Eurich et al., 2014), while 19 articles can be characterized as adopting a recursive style (e.g., Chatterjee, 2013), and only two articles adopt a parallel style (Cavalcante, 2014a; Landau et al., 2016).^1^ Finally, there seemed to be a reference to conjunctive approaches, but the form it took was relatively implicit (Baldassarre et al., 2017).

Concerning the theoretical approaches, most of the articles rely on the BM literature or BM theory, which links them to various and multiple perspectives, such as value-based perspectives (e.g., Landau et al., 2016), system perspectives (Inigo et al., 2017), or evolutionary perspectives (e.g., Amit & Zott, 2012), among others (e.g., Tesch et al., 2017). Some articles associate BMI with organizational resources and apply resource-based and dynamic capabilities views (e.g., Schindehutte et al., 2008). New BMs are coupled with changes and innovation, with some authors drawing on disruptive innovation theory (e.g., Snihur et al., 2018). Lastly, some recurrent theories are cognitive and psychological (e.g., Schneckenberg et al., 2019) or relate to literature on sustainability (e.g., Bocken et al., 2014).

The level of analysis varies between the micro-level, where the focus is on managers, CEOs, and entrepreneurs (e.g., Eppler et al., 2011), and the macro level, that is, the levels of the firm (e.g., Snihur & Wiklund, 2019) and the BM (e.g., Stubbs, 2019). A few studies examine BMI by also considering the impacts and the influence of the sector/population at a macro level (e.g., Kalkanci et al., 2019).

The articles included in our review considered several industries. However, these are mainly from the manufacturing and service sectors, and there is an emphasis on the more digitalized sectors such as information and communications technology (Khanagha et al., 2014) or banking (Dunford et al., 2010). Geographically, the sample is mainly distributed within Europe, excluding Eastern Europe but including Southern Europe (e.g., Ghezzi, 2012), Northern Europe (e.g., Aspara et al., 2013), and Western Europe (e.g., Winterhalter et al., 2017). Our review points to a lack of studies covering other geographic areas. Only two studies were recently conducted in Oceania—one each in Australia (Stubbs, 2019) and New Zealand (Islam, 2019)—and one in Africa (Habtay, 2012). Other underrepresented areas are Asia (six studies) and North America (four studies). There were no studies in South and Central America.

The specificities highlighted above delineate important aspects of this body of knowledge on BMI processes that will constitute some of the key elements we use to build our future research agenda.

## Identifying BMI processes

Although it is clear from the process-focused BMI literature that there are many ways to generate BMI, we argue this heterogeneity has not been sufficiently recognized. Indeed, research on BMI processes has expanded in multiple directions, leading to mixed and disparate conceptualizations of BMI processes and/or sub-processes that vary based on the various contexts considered. For instance, for platform business models, Andreassen et al. (2018) propose a T-model for BMI value creation processes; Cavalcante (2014a) propose linear steps for BMI development; Kiura et al. (2014) illustrate a systems-based methodology based on learning processes; Amit and Zott (2012) propose BMI processes for incumbents; and Snihur (2016) does the same but for new ventures.

To organize this heterogeneous literature and acknowledge the ontological differences among themes in a parsimonious manner, we identified five different types of BMI processes: *cognition processes for BMI, knowledge-shaping processes for BMI, strategizing processes for BMI, value creation processes in BMI,* and *evolutionary learning processes as the glue of BMI processes.* We categorized each article in our sample as dealing predominantly with one of these salient processes. The analysis shows that the BMI strategizing processes form the most populated category (47 papers), followed by value creation processes (26), generative cognition processes (17), knowledge-shaping processes (16), and evolutionary learning processes (7).^2^ We further analyze the articles and identified sub-processes of each BMI process***.***

By focusing on the nature of each of the BMI processes and how they are interrelated, our understanding of the literature leads us to locate each BMI process inside a framework (Table 1) that summarizes different sub-processes under each main process category. This framework shows that BMI processes can be represented as embedded in broader organizational contexts. More specifically, the framework includes: the context of individuals and the top management teams (where previous literature mainly studied the cognition processes) and the entire organization (or part of it) through structured patterns of action and interaction involving not only several actors and departments (at this level, previous studies focused on knowledge-shaping processes to explore and/or experiment with BMI) but also broader organizational levels (strategizing and value creation processes have been mainly studied at the level of organizations in relation with their markets and stakeholders). Moreover, the processes happen not only in the nexus of the same actors. In line with the latest studies about the concept of interplay in strategy studies (e.g., Weiser et al., 2020) and with the concept of interdependence in organization design studies (see, for instance, Raveendran et al., 2020), papers about BMI processes explore different types of processes enacted by different entities, emerging as boundary-spanning and interrelated with each other (e.g., Foss & Saebi, 2017, 2018; Frankenberger & Sauer, 2019; Snihur & Tarjizan, 2018; Teece, 2018; Zott & Amit, 2007, 2017). BMI results from a continuous process of refinement that connects individuals, teams, organization units, markets, and institutions (Andreassen et al., 2018; Forkmann et al., 2017; Inigo et al., 2017). We propose an integrative analysis of these interconnections and identify a unique general framework (Table 1) that connects all the BMI processes.

**Table 1:**
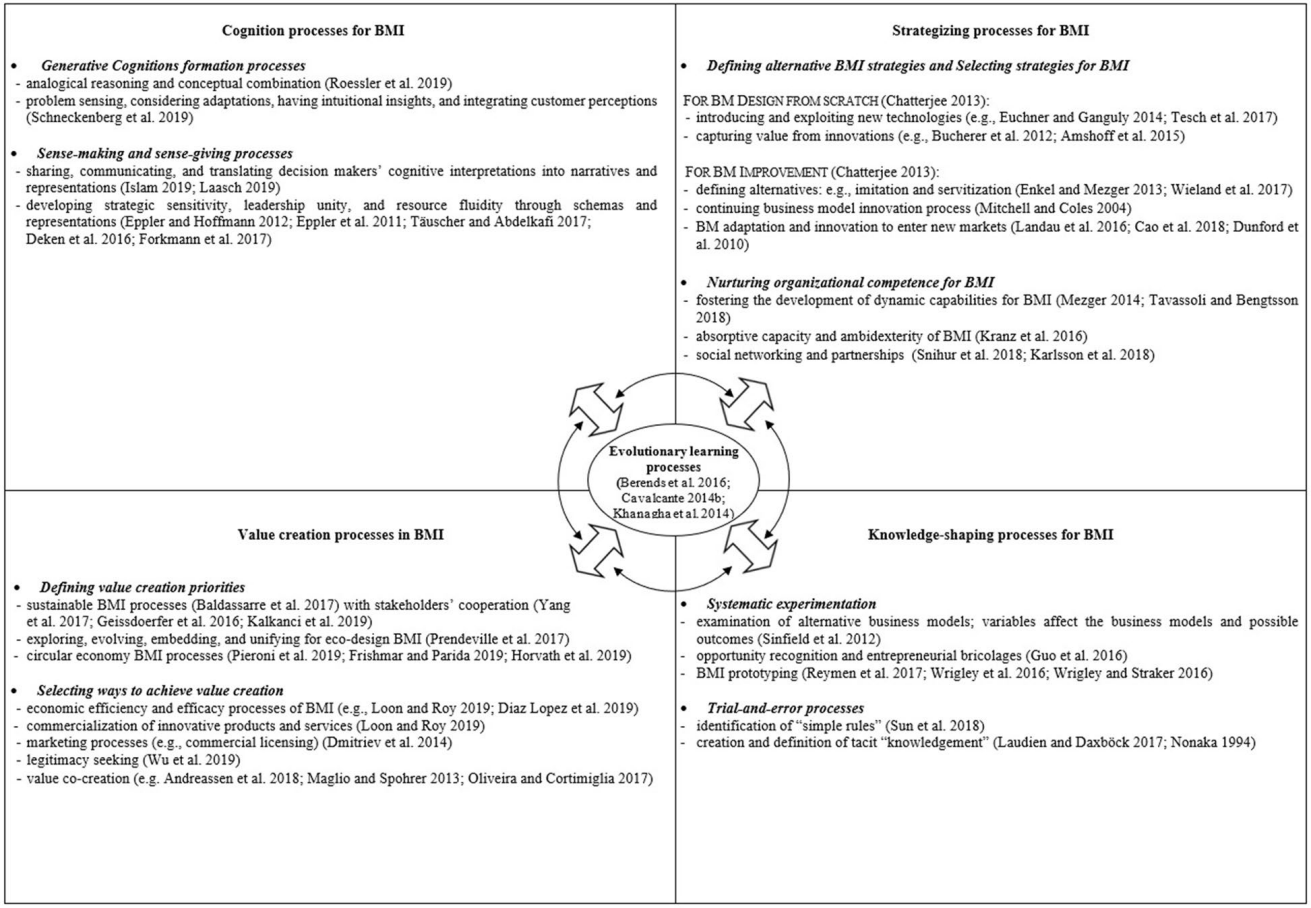
BMI processes framework and the most relevant references

### Cognition processes for BMI

Cognitive processes refer to the belief systems that are held by top managers (Aspara et al., 2011) and middle managers (Groskovs & Ulhøi, 2019) and drive and support their managerial decisions (Martins et al., 2015). Accordingly, the main focus of this cognitive perspective is the formation processes of decision makers’ mental representations, their mindsets related to the key business ideas, and their unique view of the business (e.g., Gavetti & Rivkin, 2007). The first sub-process we identified is the generative cognition process, which relates to all the papers investigating how decision makers’ belief systems, psychology, and mindsets form and are shared inside organizations to generate new ideas for BMI (Roessler et al., 2019). The BMI literature suggests that *analogical reasoning* and *conceptual combination* are considered the central generative cognition processes behind BMI development (Roessler et al., 2019). In particular, analogical reasoning represents the “correct identification of similarities between an existing business model and an analog concept and the appropriate transfer of attributes and relationships from the analog to inform the activity system of the target business model” (Martins et al., 2015, p. 109), while conceptual combinations are the “creation of new concepts that are variants of existing ones” (Martins et al., 2015, p. 111) in business model design.

The generative cognitive processes have to be applied in the contexts in which the managers and entrepreneurs work. Accordingly, Schneckenberg et al. (2019) identify context-based cognitive processes for business model innovation (i.e., *problem sensing*, *considering adaptations*, *intuitional insights*, and *integrating customer perceptions*) that support an emerging logic in business model design. In particular, problem sensing relates to cognitions stemming from decision makers’ frustration and disappointment when customer needs are not met as the managers intended; considering adaptations involves the decision makers’ ability to match rational cognition processes with collaboration encouragement, open-mindedness, and risk-taking; intuitional insights are cognitive processes related to intuition and having a clear vision of BMI configuration for the company; and finally, integrating customer perceptions relates to a profound and genuine interest in and consideration of consumers’ needs and expectations (Schneckenberg et al., 2019). The cognitive approach is also applied in the entrepreneurship literature. For instance, Velu and Jacob (2016) focus on entrepreneurs’ leading role in designing BMI, while Roessler et al. (2019) focus on the constraints and limitations of entrepreneurs’ cognitive capabilities, which could be associated with previous successes. All these cognition sub-processes are particularly significant for the imprint that entrepreneurs leave on their ventures (Snihur & Zott, 2020). More specifically, imprinting processes are a set of cognitive practices that entrepreneurs enact to give novel structures to ventures and explain how BMI emerges and persists.

In sum, this first batch of papers addresses the generative cognitive processes developed by managers, entrepreneurs, and top management teams to generate BMI (Olofsson et al., 2018; To et al., 2018).

The second batch of papers adopts a cognitive approach to BMI and relates to the sense-making and sense-giving processes that managers, entrepreneurs, and top management teams have to tackle when developing or introducing new ideas for a business model in an organization. The sense-making and sense-giving processes follow the decision makers’ generative cognitive processes, which have to be shared, communicated, and translated into narratives and representations to enact any kind of BMI (Islam, 2019). The interpretive perspective helps to explain how BMs can be changed and innovated through representations that make sense of things and create a common and communicable understanding of how organizations create, change, innovate, and exchange value on the market. This can happen through artifacts, such as annual reports, BM representations, and other representations that managers use to translate, negotiate with, and reconcile multiple stakeholders (Laasch, 2019). Schemas or cognitive representations are extremely important for sense making and sense giving regarding BMI generation (Eppler & Hoffmann, 2012; Eppler et al., 2011; Täuscher & Abdelkafi, 2017). These cognitive representations are useful tools for managers to develop strategic sensitivity in a company, develop leadership unity, and provide resource fluidity (Deken et al., 2016; Forkmann et al., 2017).

In this regard, many authors have found tight interconnections between cognitive processes for BMI and strategizing processes (Aspara et al., 2011; Brink, 2017; Schindehutte et al., 2008); between cognitive processes for BMI and value creation (Laasch, 2019; Inigo et al., 2017; Maglio & Spohre, 2013); and knowledge-shaping processes (Berends et al., 2016; Cavalcante, 2014a, b; Forkmann et al., 2017). These interconnections change according to the theoretical approaches adopted by the researchers and the particular objective of the paper. According to our systematic literature review, many authors emphasize the interrelations between cognitive processes and strategizing processes. Most of this research focuses on the translating mechanisms that companies and managers develop to transfer cognitive processes into strategic designs and processes, which is essential to better understanding the feasibility of the ideas and their ability to create value (de Oliveira & Cortimiglia, 2017; Storbacka et al., 2013; To et al., 2018).

### Strategizing processes for BMI

Translating the managers’ cognitions into firm-level strategizing processes means transforming ideas to enact BMI in specific contexts (Roessler et al., 2019). Strategizing processes involve setting and maintaining companies’ competitive advantage on the market (Casadesus-Masanell & Ricart, 2010; Demil & Lecocq, 2010; Magretta, 2002; Osterwalder et al., 2005; Porter, 1985; Teece, 2010). Through our ontological analysis, we identified three different strategizing processes that companies enact in relation to BMI, namely, defining alternative BMI strategies, selecting strategies for BMI, and nurturing organizational competence for BMI.

When designing BMI, the process of defining alternative BMI strategies determines organizations’ direction and presence on the market (Broekhuizen et al., 2018; Martin‐Rios & Parga‐Dans, 2016). There are many BMI alternatives that decision makers can choose from. According to our systematic literature review, the most investigated are: imitation and replication (Enkel & Mezger, 2013), a customer-centric business model (Pynnönen et al., 2012), and servitization (Wieland et al., 2017). Imitation relates to the strategy adopted by followers who imitate the entire or part of the business model innovation adopted by competitors and benefit from the errors and difficulties that first-movers have to overcome when introducing innovation into organizations and onto the market (Semadeni & Anderson, 2010). Customer-centric strategies emphasize market research, marketing, channels, and customer relationship processes as the main competitive advantage (Sheth et al., 2000). By contrast, servitization concerns the shift from a product-centric activity to a service-dominant logic, where institutional change processes are facilitated to identify and develop new forms of business models (Naor et al., 2018; Sjödin et al., 2020; Storbacka et al., 2013).

However, the BMI alternatives should reflect the organizational resources, the industry, the markets, and the firms’ competitive landscape. Accordingly, when the pathways to BMI are not clear, decision makers have to decide whether to undertake a BMI design process from scratch (i.e., create new business models) or embark on a BMI improvement process (i.e., develop existing business models), which usually happens at later points in the ideation and exploration processes (Cavalcante, 2014a; Cortimiglia et al., 2016; Eurich et al., 2014; Kiel et al., 2017; Landau et al., 2016; Parmentier & Gandia, 2017; Remane et al., 2017; Winterhalter et al., 2017).

When decision makers opt for BMI design from scratch, they have to activate an iterative process that includes identifying the firm’s business category and a series of interrelated choices that convert the generic value-capture logic of a business model into firm-specific and measurable core tasks. This process concludes with a detailed map of the activity system needed to achieve the core firm’s objectives (Bucherer et al., 2012; Chatterjee, 2013; Euchner & Ganguly, 2014). The creation of new business models from scratch is mostly related to the introduction and exploitation of new technologies (Amshoff et al., 2015), such as Internet of Things technologies (García-Gutiérrez & Martínez-Borreguero, 2016; Tesch et al., 2017) and digitization (Jensen & Sund, 2017; Vasarhelyi & Alles, 2008). However, the decision to use a BMI design from scratch is often determined by market-driven disruptive approaches to the market (Habtay, 2012).

When companies opt to develop existing business models, whatever the type of BMI change (e.g., business model extension, revision, or termination), managers should abandon repetitive goal-oriented activities related to specific business models (Cavalcante et al., 2011) to introduce the “continuing business model innovation processes,” a never-ending process of creation, introduction, change, and development of different types of innovations that continue over time and do not stop once the business model has been implemented (Mitchell & Coles, 2003, 2004). To execute this perpetual process, some authors identify a set of computer-based tools that can help the project team identify areas in BMs to improve or change (Ebel et al., 2016). Others suggest different processes according to the contexts in which the companies are embedded. In this vein, Ammar & Chereau (2018) suggests different paths to innovate the business model components in small and medium enterprises, while other authors develop models of BM adaptation and innovation to enter new markets (Cao et al., 2018; Dunford et al., 2010; Landau et al., 2016). Stubbs (2019) focuses on BMI strategies related to socially responsible companies, and Bogers et al. (2015) examine BMI strategies for family-owned companies.

From a strategic point of view, BMI requires firms to develop new *resources and competences* at the organizational level (Amit & Zott, 2001; Demil & Lecocq, 2010; Mezger, 2014). Using our ontological analysis, we identified the processes nurturing organizational competences for BMI. Nurturing organizational competence for BMI means supporting dynamic capabilities, absorptive capacity, ambidexterity, social and business networking, and partnerships. For BMI, dynamic capabilities (Demil & Lecocq, 2010), absorptive capacity, and ambidexterity (Kranz et al., 2016) are especially important. Dynamic capabilities are defined as the skills to (re)configure resources and routines to adapt to changing markets and the business environment (Teece, 2007). Building on Teece (2007), Mezger (2014) discusses how to develop dynamic capabilities through sensing, seizing, and reconfiguring processes. Absorptive capacity is defined as the skills of the organization that allow it to recognize and incorporate knowledge from external markets/environments and use this knowledge to reconfigure the organization. Organizational ambidexterity is the firm’s ability to both properly manage an existing business model and develop dynamicity when facing changes in the market (Kranz et al., 2016; Schindehutte et al., 2008). Strategically, other organizational competences that organizations have to enact to facilitate BMI are social networking and partnerships (Karlsson et al., 2018; Snihur et al., 2018)*.* They are based on an organization’s ability to enter and interact in dynamic business and social networks and ecosystems (Ghezzi et al., 2010; Snihur et al., 2018).

Therefore, we conclude that the two different strategizing processes (defining and selecting alternative BMI strategies and nurturing organizational competence for BMI) are interrelated and involve various actors across different levels of the organization.

Moreover, strategizing processes are interrelated with value creation processes (Bogers et al., 2015; Cao et al., 2018; Chatterjee, 2013; Ghezzi, 2012; Kiel et al., 2017; Landau et al., 2016; Storbacka et al., 2013; Wieland et al., 2017). As we explain in the following section, this relationship can be driven and/or mostly mediated by knowledge processes, such as experimentation and trial-and-error (e.g., Baldassarre et al., 2017; Forkmann et al., 2017; Laïfi & Josserand, 2016).

### Knowledge-shaping processes for BMI

Only having cognition and strategizing processes is not enough to develop BMI. The literature highlights the importance of testing BMI and disseminating the underlying knowledge throughout the entire organization (Axelson & Bjurström, 2019; McGrath, 2001). Knowledge processes refer to the processes that the departments, divisions, and teams within an organization can use to generate innovative ideas and innovations (Nonaka, 1994). Accordingly, knowledge processes are social processes that mainly occur at the team level and are considered the main source of a firm’s competitive advantage (Fiol & Lyles, 1985; March, 1991). Moreover, unlike product innovations, BMI is not limited to production or R&D departments but involves interdepartmental and multifunctional teams (Malhotra, 2002; Sinfield et al., 2012). Thus, knowledge-shaping activities for BMI requires dedicated teams that aim to explore and experiment with ideas and innovative solutions to enhance BMI (Berends et al., 2016). According to our ontological analysis, there are two main knowledge processes: systematic experimentation and trial and error.

#### Systematic experimentation

Maintaining a portfolio of experimentations of new business models, even when BMI is settled (e.g., experimenting with alternative sales channels, testing servitization or the more recent platformification,^3^ and even exploring alternative target customers/segments), has proved to be effective against a crisis like COVID-19 (Andries et al., 2013). In the same vein, experimentation is a vital activity when high-speed innovation is of great concern (Tuulenmäki & Välikangas, 2011; Wrigley & Straker, 2016; Wrigley et al., 2016). Experimentation means testing hypotheses and assumptions by using specific plans and procedures (Reymen et al., 2017). Unlike trial-and-error processes, experimentation is grounded in science, usually preceded by an analytical phase, and followed by specific plans and procedures. The business model experimentation process starts with an examination of alternative business models, the variables affecting the business models, and the possible outcomes (Sinfield et al., 2012). Then, different experimental analyses allow the firm to narrow its choices and pursue the business model that benefits the company the most. An activity that facilitates the experimentation processes is BMI prototyping, which starts by identifying the value proposition for a specific market segment, then evaluates the business model components, and finally constructs the BM alternatives. Through a cause-effect procedure, decision makers choose the alternative BM that can best reduce technological uncertainty and maximize profits (Reymen et al., 2017). Routine-based activities like opportunity recognition (i.e., actions in identifying opportunities) and entrepreneurial bricolages (i.e., applying combinations of the resources at hand to new problems and opportunities) can also facilitate experimentation (Guo et al., 2016).

*Trial-and-error processes*, by contrast, relate to a knowledge-shaping activity without a formal and explicit plan (Enkel & Mezger, 2013; Laudien & Daxböck, 2017; Sosna et al., 2010) and sometimes even through unintended activities. For instance, Sun et al. (2018) demonstrate that business model innovation develops in a way that reflects entrepreneurs’ early experiences (e.g., through feedbacks and reactions) and enables the emergence and identification of the so-called (often tacit) “simple rules” that gradually form the basis for tentative or temporal new business models development. Trial-and-error processes are the most common ones through which companies create the tacit knowledge necessary to develop BMI (Laudien & Daxböck, 2017; Nonaka, 1994).

In sum, knowledge shaping generated through processes that are unintentional and/or based on decision makers’ experience and those that are the result of planned, test-driven activities involving actors across the organization are two different but not mutually exclusive processes that can be used to explore the production of BMI.

Many authors have emphasized that knowledge shaping (i.e., experimentation and trial-and-error) is an essential part of BMI because it is recursively interconnected with strategizing and cognitive processes (e.g., Cavalcante et al., 2011; Chesbrough, 2010; Demil & Lecocq, 2010). A topical example is the circular economy, which has become a fertile research context for BMI experimentation, where the knowledge-shaping process is investigated along with strategizing and cognitive processes (Konietzko et al., 2020; Lopez-Nicolas et al., 2020; Baldassarre et al., 2020).

### Value creation processes in BMI

The final goal of engaging in BMI is to create value for organizations and their stakeholders. The value creation processes are sets of activities that enable companies and stakeholders to realize their own value from BMI (Zott & Amit, 2007; Amit & Zott, 2012). However, the focus on the type of value and how companies create value has been subject to debate in the literature. We have divided the BMI process-based literature into two different streams. The first is *defining value creation priorities*, which is a stream about the different types (economic, social, and environmental) of values that are a priority when companies create and develop BMIs in actual markets. The second category of papers includes the processes of *selecting ways to achieve value creation*, that is, how companies produce value for their organizations when engaging in BMI.

Defining value creation priorities in BMI mostly refers to the literature on sustainable BMI. Sustainable BMI processes are defined as innovative ways that have a significant positive and/or significantly lower negative impact on the environment and/or society because of changes in the way the organization delivers and captures (economic) value (Baldassarre et al., 2017). Accordingly, these BMIs incorporate triple-bottom-line priorities and consider a wide range of stakeholder interests, including society and the environment. This means that companies should follow specific processes and a value proposition design aimed at understanding the stakeholders’ needs and interests, finding problem-fit solutions, and testing the product in cooperation with the stakeholders (Geissdoerfer et al., 2016; Kalkanci et al., 2019; Yang et al., 2017). In the same vein, Prendeville et al. (2017) introduce a conceptual framework that, through four key transitional phases (exploring, evolving, embedding, and unifying), can support companies creating new sustainable business value. For example, circular economy BMI has recently emerged and may have greater appeal to stakeholders and generate greater profit for companies than sustainable BMI (e.g., Pieroni et al., 2019; Frishmar & Parida, 2019; Horvath et al., 2019). Finally, sustainable BMI can change according to specific industries like manufacturing (Short et al., 2014), a manufacturer of original automotive equipment (Spieth et al., 2019), newspapers (Günzel & Holm, 2013), and tourism (Alegre & Berbegal-Mirabent, 2016).

The BMI process entitled *selecting ways to achieve value creation* started with the advent of BMI literature (Euchner & Ganguly, 2014; Leavy, 2010; Simmons et al., 2013) and continues to be a hotly debated topic. BMI is a resource-demanding and expensive activity; for this reason, finding ways to make innovative BMs profitable, exploiting opportunities, and reducing costs are compelling issues for managers, as well as researchers (Loon & Chik, 2019; Diaz Lopez et al., 2019a, b). One of the most-cited processes to enable BMI is the commercialization of innovative products and services (Loon & Chik, 2019); in the same vein, Dmitriev et al. (2014) focus on marketing processes (e.g., commercial licensing) that allow companies to capture value by commercializing technologies, thereby driving continuous business model innovation; while in service industries, the pay-per-use business model is becoming a popular way to capture value from the market (Naor et al., 2018). In their investigation of hybrid organizations whose business models blur the boundary between for-profit and nonprofit operations, Alberti and Garrido (2017) underline the importance of these organizations generating profits from their own resources instead of exploiting external resources that could generate the highest profits. Another aspect to consider is legitimization, which is especially important for disruptive and innovative BMs (Wu et al., 2019).

Finally, since value delivery for stakeholders is one of BMI’s most important goals, another way to achieve value creation in BMI is to co-create it with and for multiple stakeholders (Andreassen et al., 2018; Angeli & Jaiswal, 2016; Maglio & Spohrer, 2013; Oliveira & Cortimiglia, 2017; Tolkamp et al., 2018). Most of these studies propose triadic business models, where value is co-created by suppliers, buyers/users, and companies (Andreassen et al., 2018). For these business models, value co-creation processes should be supported by knowledge, skills, and collaboration, and it is also essential to understand how to allocate resources among the actors involved (de Oliveira & Cortimiglia, 2017).

In sum, value creation processes for BMI—more so than all the other BMI processes—involve actors across a wide variety of levels spanning boundaries and including not only organizational but also external actors (stakeholders, customers, etc.). This process is the end but also the starting point for the BMI processes. According to the value created, co-created, and captured, BMI can be fine-tuned, changed, and innovated to activate cognitive, strategizing, and/or knowledge-shaping processes (Frishammar & Parida, 2019; Snihur & Wiklund, 2019).

### Evolutionary learning processes as the glue of BMI

Van de Ven and Poole (1995) define evolutionary processes as a sequence of variation, selection, and retention of events among entities in competitive contexts with scarce resources. Similarly, BMI processes, which are varied, selected, and then retained, aim to gain a competitive advantage over competitors to guarantee their own existence in the market and can be considered evolutionary as well. An unexpected result of our analysis is the role of learning activities within and between each BMI process. For instance, learning processes are related to updating beliefs; if this relationship does not occur, it can impede BMI (Nailer & Buttriss, 2020). In strategizing processes, even imitation needs learning processes to change and adapt existing BMs (Sinfield et al., 2012; Zhara et al., 2006). In the same vein, knowledge and learning processes are different processes but strictly correlated, since the former are made by functional and technical process aimed at testing and creating prototypes and experimentation for BMI, while the latter (learning processes) relate to the acquisition of new knowledge (Cavalcante, 2014b; Kiura et al., 2014; Thurner et al., 2019). Finally, value creation processes make it possible to gain useful information from the market to fine-tune the value proposition of BMI (Spieth et al., 2019; Simmons et al., 2013). Moreover, learning processes appear to be an effective connector between two or more BMI processes (Balocco et al., 2019; Cavalcante, 2014a, b; Enkel & Mezger, 2013; Nailer & Buttriss, 2020; Sinfield et al., 2012). For instance, learning processes can link cognition, knowledge-shaping processes, and strategizing processes for BMI (Cavalcante, 2014b). Strategy formulation processes are tightly intertwined with learning processes, as the formulation and identification of different strategic alternatives can be used as a collective learning experience, especially when the innovation of existing business models is a priority (Khanagha et al., 2014). These findings are in line with the BMI process literature that is explicitly and/or implicitly interested in evolutionary learning and, based on feedback regarding actions and trials driven by decision makers’ beliefs and mindsets, considers BM innovations as the outcomes of continuous learning processes (Chesbrough, 2010; McGrath, 2001; Sosna et al., 2010; Martins et al., 2015).

As shown in Table 1, we define *evolutionary learning* processes as the glue of BMI processes. Evolutionary learning processes are characterized by different learning episodes that occur when various BM components evolve (Berends et al., 2016) and engage *“stakeholders continually in ‘learning’ how to better deal with the complex issues they are facing over time”* (Kiura et al., 2014, p. 698).

Thus, we envision evolutionary learning processes as successive, persistent, and circular processes that connect cognitive and shape knowledge, strategizing, and value creation processes.

## Discussion and research agenda

In this section, we first discuss the findings related to the interconnections between different categories of BMI processes. In line with process theory, which considers phenomena as evolving and interrelated events enacted by entities (Nailer & Buttriss, 2020; Rescher, 1996), we identified patterns of processes to understand how BMIs emerge, develop, grow, or come to an end.

We argue that this view has great potential for advancing and enriching research on BMI processes because it resonates particularly well with the emerging work on such topics and with process research in general (Langley et al., 2013; Cloutier & Langley, 2020). We then consider how this literature has developed, along with various theoretical perspectives, and propose a research agenda that will advance BMI process theory by trying to address the research gaps we identify.

### Discussion

Our review aimed to synthesize the research on BMI processes. At least two relevant contributions can be seen here. First, we clarify the BMI process construct by developing a BMI process framework with a categorization of different types of BMI processes (see Table 1) and explaining how they are different from the related sub-processes. Second, by developing a unifying framework, we identify five BMI processes and show that they are distinctive yet interconnected and interrelated. A key feature of BMI processes is that they are enacted both *within* and *across* different levels. Thus, BMI processes imply a multitude of interactions among actors on the same level (e.g., actors belonging to the TMT or a specific business unit, department, etc.) or across these levels (e.g., actors from a business unit with actors from the TMT, entrepreneurs with actors from the R&D function, etc.). Some authors prefer to focus on actors *within* the same level of analysis, such as teams of managers (e.g., Eppler & Hoffmann, 2012) or entrepreneurs (e.g., Reymen et al., 2017); this happened mainly when the main objective was to describe specific generative cognition processes or knowledge-shaping processes (Deken et al., 2016; Sinfield et al., 2012). By contrast, other scholars focus on actors *across* different levels. For example, Aspara et al. (2011) show the embeddedness of cognitive processes in strategic ones, which implies an interplay of actors *across* different levels and reveals that TMT and individual cognitions (personal values, beliefs, and backgrounds) shape the selection of appropriate strategies (TMT level) and, eventually, the BMI (firm level).

Thus, the five BMI processes that we have identified are interconnected and enacted by actors within and across different levels of the organization. This key feature probably explains why, in our analysis, we could not identify any specific theoretical framework guiding the bulk of the papers. While no theory showed significant prevalence, we could still identify some emerging patterns. Specifically, papers focused on cognition processes tended to be mostly grounded in cognitive-psychological perspectives (Roessler et al., 2019; Schneckenberg et al., 2019; Täuscher & Abdelkafi, 2017) and considered, for example, structure-mapping theories and theories of pattern recognition (Roessler et al., 2019). In such cases, they operated mainly within levels of analysis such as managers (e.g., Schneckenberg et al., 2019) or entrepreneurs (e.g., Roessler et al., 2019). Papers focused on value creation processes tended to put more emphasis on the sustainability literature (e.g., Bocken et al., 2014; Frishammar & Parida, 2019; Kalkanci et al., 2019; Pieroni et al., 2019; Yang et al., 2017) than the other papers.

It was interesting to note that some of the knowledge-shaping processes and strategizing process papers explicitly named the BMI literature (Broekhuizen et al., 2018; Chatterjee, 2013; Remane et al., 2017) or business model theory (Balocco et al., 2019; Broekhuizen et al., 2018; Laasch, 2019; Landau et al., 2016; Short et al., 2014; Viswanadham, 2018) rather than use more traditional and well-established theories. More specifically, knowledge-shaping processes papers tended to couple the BM literature with innovation issues, for example, by studying new technology-based ventures (e.g., Balocco et al., 2019; Reymen et al., 2017) or including aspects of open innovation (e.g., Huang et al., 2013) and knowledge management (Malhotra, 2002). Papers related to strategizing processes dealing with BM design from scratch tended to couple the BM literature with some other theories (e.g., disruptive innovation theory (Habtay, 2012; Snihur et al., 2018). Finally, papers related to strategizing processes dealing with BM improvement tended to connect the BM literature with dynamic capabilities theory (Mezger, 2014), RBV (Pynnönen et al., 2012), or a combination of the two (Schindehutte et al., 2008).

This approach of relying on the business model literature or BM theory rather than extant, more established theories was quite consistent in our sample of papers and a good signal that the time is probably ripe for the emergence of a process-based BMI theory that could leverage the richness of empirical research findings to explain how and why BMIs “emerge, develop, grow, or terminate over time” (Langley et al., 2013, p. 1). To this end, the following section offers some insights for future research.

### Insights for future research

Drawing on two key features of our BMI process framework (Table 1), namely the interconnectedness and the multifaceted nature of BMI processes, we focus on the emergence of two main research directions and point to related opportunities for future research.

#### Expanding research on the interconnection of BMI processes

The first research direction refers to the expansion of research on the interconnection of BMI processes. Our review shows that BMI processes are interrelated and involve different actors across different levels of the organization, and most of the papers in our review consider two or more process types simultaneously. BMI processes are attracting increasing research attention, and there has been extensive debate concerning what the term “process” should imply from a theoretical point of view (see Langley et al., 2016; Van de Ven & Poole, 1995) and from a research design point of view (see Langley et al., 2013). A key point of this debate, which is critical to advancing BMI process studies, is the distinction between what has been called ‘weak’ and ‘strong’ process theory (Langley et al., 2016). Weak process theorizing typically incorporates the concept of change and evolution over time but regards processes as events ‘happening to things’ that maintain their unique identity over time. So, for example, with a few exceptions (Broekhuizen et al., 2018; Snihur, 2016) in BMI research, many studies have theorized about and analyzed the change in an organization’s business model over time while assuming that the organization preserves its essence throughout the process.

By contrast, applying a ‘strong’ process ontological perspective means viewing the BM as a dynamic bundle of qualities (Langley et al., 2013) and all the observed elements and actors of BM as “momentary instantiations of processes” (Cloutier & Langley, 2020, p. 3). Of course, there is no single recipe for developing informative BMI process theories, conceptualizations, and/or analyses. Our BMI process framework (see Table 1) suggests the application of a “strong” process ontological perspective where BMI is understood as the way with which a complex reality, consisting of multiple actions and interactions, is continuously brought into being.

The problem is that when studying two or more processes simultaneously, scholars have often tended to assume that processes are linked in a merely consequential manner. For example, it was assumed that cognitive processes come before knowledge-shaping BMI processes (e.g., Forkmann et al., 2017) and not the other way around. Although this consequential approach has proved useful, considering more complex links—and, in particular, the fact that all the different types of processes we have identified are all virtually interconnected—may enrich our understanding of the phenomenon (Nailer & Buttriss, 2020; Rescher, 1996). Thus, we see a need for future research to sharpen its focus in this direction. Good practices have started to highlight interesting patterns, for example by showing how cognition processes lead to value creation through knowledge-shaping processes for BMI or through strategizing processes (Broekhuizen et al., 2018).

Starting from here, future empirical research may aim to fully validate our BMI process framework (Table 1). It would also be interesting to better understand what the most relevant relationships among the different process types are and how we could observe the interactions among different processes. Finally, our review points to learning processes as particularly important connectors/glue among the various processes. Future research should further test this finding and explore the presence of other connectors. In other words, other than learning, are there any connectors linking the different BMI processes? Which key relationships among different processes determine BMI success or failure? And how could we observe the interaction among different processes?

#### Embracing more complex process theorizing styles

The variegated nature of BMI processes that has clearly emerged in our review suggests a need to consider different process theorizing styles. Indeed, whether a study uses linear, recursive, parallel, or conjunctive styles can significantly change its ability to contribute to the understanding of BMI (Cloutier & Langley, 2020). Unfortunately, scholars have tended to overlook these considerations in their contributions regarding BMI processes, and when they did, BMI processes were often presented through linear models that overlooked the possible presence of recursive and conjunctive processes.

A problem with linear models is that they typically rely on stage-based patterns that oversimplify the multiplicity of interactions occurring within and across different actors and fail to reveal the explanatory mechanisms that produce such patterns (Van de Ven, 1992). In addition, scholars adopting linear approaches tend to focus more on describing the steps to follow to reach the desired outcome (i.e., innovating the business model) than on explaining what might happen after a BMI process has been carried out (see Cloutier & Langley, 2020). For this reason, we encourage scholars to accept the intrinsic complexity of BMI processes and draw more on recursive and conjunctive approaches.

Using recursive approaches to BMI processes would mean that they continuously adapt via feedback loops. This approach to theorizing and analyzing BMI processes has the advantage of applying to many BMI process phenomena and allowing for different contexts to be considered, including multiple levels of analysis. Recursive approaches, unlike their linear counterparts, embrace a more processual ontology where “phenomena are embedded in social interactions, continually changing and mutually constituting each other across levels and over time” (Clourtier & Langley, 2020, p. 4). Building on this approach, future research may get a better understanding of the role of feedback loops in BMI processes. Some of the papers we have analyzed are moving in this direction: For instance, by adopting a user-centered approach, Tolkamp et al. (2018) show that the interaction between multiple levels (in this case, firm and market levels) generates an involvement loop between the user and the firm in the BM design process that facilitates BM adaptation and can lead to incremental and radical BMI thanks to user feedback. Another example is provided by Groskovs and Ulhøi (2019), who see BMI as an iterative, dynamic, and continuous process of search and change activities. They highlight the importance of cognition processes deriving from middle managers who sense the environmental dynamics and strategically help the CEO and senior management to allocate resources amid continuous change and to make corrections according to the iterative loop cycles.

Some of the interesting questions that we can ask in this regard include: When are feedback loops adjustive and when are they generative for BMI? What are the feedback loops that generate incremental BMI processes, and which loops contribute to disruptive ones? How can multiple actors’ interactions be depicted in BMI processes?

In the same vein, we encourage future studies of BMI processes to explore the application of conjunctive approaches to BMI processes. This would imply that scholars make connections between diverse elements of BMI and deliberately go beyond the distinctions inherent in the BMI process literature that have often led previous scholars to see different types of BMI processes in a rather compartmentalized manner. The numerous bidirectional and circular arrows in our categorization (Table 1) are meant to acknowledge the possibility of conjunctive approaches to BMI processes. Thus, we invite future studies to deepen these intuitions. For example, Baldassarre et al. (2017) have applied a conjunctive approach to develop a process for sustainable value proposition design combining sustainable BMI and user-driven innovation, revealing that multiple BMI processes interact with each other several times and in various ways in different stages of BMI. Following this direction may entail asking: How can the use of a conjunctive approach to BMI processes foster the emergence of a distinctive BMI theory? What are the most relevant actors and structures for an understanding of BMI processes via a conjunctive approach? How can a conjunctive approach facilitate the simultaneous consideration of multiple BMI processes?

In conclusion, our review has shown that BMI processes, like many others, are typically relational (e.g., Laasch, 2019), temporal (e.g., Schneckenberg et al., 2019), and situated (e.g., Snihur & Wiklund, 2019) and require interpretative open-endedness (e.g., Villani et al., 2017). We are confident that accounting for the interconnectedness and the multifaceted nature of BMI processes will make it possible to advance our knowledge while preserving these features of BMI processes as much as possible (Tsoukas, 2017).

### Conclusion

In this article, we systematically reviewed the literature on BMI processes and built a BMI process framework that involves five different but interrelated types of BMI processes. Our categorization and its application to the existing body of BMI research help integrate the various views of BMI processes into a better understanding of a multifaceted construct.

Based on our categorization and framework derived from our literature review, we provide a focused set of suggestions for future research. Research on BMI processes has blossomed over the past few decades, but there are still many opportunities for researchers to engage more fully with BMI process–based research from a theoretical and a methodological standpoint. We hope our efforts will stimulate further investigation to reach a better understanding of this important phenomenon.

## Appendix 1: Procedures for sourcing, searching, selecting, and excluding

A. Source of information
  1. Peer-reviewed journal articles only
  2. Empirical, conceptual, and review articles only
  3. English only
B. Exclusion criteria
  1. Studies focused on business model rather than business model innovation
  2. Studies including business model innovation in the title and in the abstract but where BMI was not the primary focus
  3. Research published in edited books and conference proceedings
  4. Editorials
  5. Articles discussing business model education or research techniques
  6. Case studies for teaching purposes
  7. Articles not available from the databases
C. Search method – Keyword research
  1. Articles across academic journals published until December 2020 (with no initial time boundaries) limited to scholarly peer-reviewed journals in English language
  2. Database selection using Ebsco (incorporating Business Source Premiere)
  3. Initial focus on: (a) abstract, and (b) title
  4. Keywords for search
    **Table A1:** Keyword search results
    Keywords used	TOT
    Search in AB or TI: ((“business model innovat*” OR “innov* business model”) AND (“Process*”)) ((“business model chang*” OR “chang* business model”) AND (“Process*”)) ((“business model novel*” OR “novel* business model”) AND (“Process*”)) ((“business model advanc*” OR “advance* business model”) AND (“Process*”)) ((“business model enhanc*” OR “enhance* business model”) AND (“Process*”)) ((“business model renew*” OR “renew* business model”) AND (“Process*”)) ((“business model transform*” OR “transform* business model”) AND (“Process*”)) ((“business model develop*” OR “develop* business model”) AND (“Process*”)) ((“business model experiment*” OR “experiment* business model”) AND (“Process*”)) ((“business model evolution” OR “evolution business model”) AND (“Process*”)) ((“business model upgrad*” OR “upgrad* business model”) AND (“Process*”)) ((“business model progres*” OR “progres* business model”) AND (“Process*”)) ((“business model new*” OR “new* business model”) AND (“Process*”)) ((“business model design” OR “design business model”) AND (“Process*”)) ((“business model revolut*” OR “revolut* business model”) AND (“Process*”)) ((“business model creat*” OR “creat* business model”) AND (“Process*”))	345
    Total	345
    Exclusion of non-ABS Journals	− 90
    *TOTAL PAPERS*	255
    Number of papers excluded	− 141
    Final sample	114
  5. Deleting articles published on journals that were not in the *ABS Journal Guide 2018*.
D. Search method – paper selection
  1. The authors (in pairs) read the abstracts and the introductions of all the papers (n = 255), dividing them into A, B, or C categories (A papers were relevant to the objective of the research, B papers were studies whose relevance was not clear, and C papers were not relevant).
  2. After reading the papers, the two authors compared and reconciled their categorizations.
  3. The third author re-assessed any articles excluded by one author but included by the other.
  4. All the A papers were re-checked to verify their exclusion from this category (21 papers were removed from the A category).
  5. Final check by two authors to verify the match between papers’ content and the objectives of the systematic review (in A category).
  6. Only A papers considered for the thematic analysis (n = 114).

## Appendix 2. Procedures for thematic analysis and ontological organization

A. Theme identification
  1. Two researchers individually analyzed each paper for objective, research questions, key arguments, research methods, business model process definition, business model innovation process definition, theoretical perspective, and outcomes.
  2. Individually, the researchers wrote a statement describing the primary focus of each paper and paying attention to the conceptual terminology and vocabulary used by the authors.
  3. After 30 papers, the researchers compared their statements and discussed how to resolve any misalignments.
  4. The statements were used to enable the identification of five thematic categories of processes.
  5. Preliminary names were given to the thematic categories.
  6. Definitive category names resulted from discussions and interactions between authors, and these thematic categories were applied to the remaining papers in the Excel file.
  7. Every 30 papers, the researchers aligned their results for consistency.
B. Ontological organization, interpretation, and validation
  1. The authors discussed and agreed on the five thematic process categories (cognition processes, knowledge-shaping processes, strategizing processes, value creation processes, and evolutionary learning processes) for each paper.
  2. The authors reviewed and checked for redundancy or duplication.
  3. For each theme, the authors wrote an explanation to check the fit between the content of the paper and the themes, assuring ontological consistency.
C. Ontological organization, interpretation, and validation
  1. The authors discussed and agreed on the five thematic process categories (cognition processes, knowledge-shaping processes, strategizing processes, value creation processes, and evolutionary learning processes) for each paper.
  2. The authors reviewed and checked for redundancy or duplication.
  3. For each theme, the authors wrote an explanation to check the fit between the content of the paper and the themes, assuring ontological consistency.
D. Quality checking
  1. Each paper was codified independently by two researchers who paid the same attention to each.
  2. The process was thorough, inclusive, and comprehensive (three thematic descriptors).
  3. The interaction process implied a comparison of the selected themes going back and forth to the original papers.
  4. The authors checked for internal coherence, consistency, and distinction.
  5. The authors interpreted the papers by using their own meanings and maintaining the vocabulary expressed in the papers as much as possible.
  6. Data and themes were paired iteratively.
  7. The authors used ontology tables for consistency.
  8. The authors played an active role in each phase.

## Footnotes

1 For the remaining articles, we were not able to code them in one of the aforementioned BMI process approaches.

2 One paper was not categorized because it is a literature review.

3 The term platformification was first introduced by Shevlin (2016), who defines it as “a plug-and-play business model that allows multiple participants (producers and consumers) to connect to it, interact with each other, and create and exchange value)”.
